# The effects of different music types on tennis performance among recreational players

**DOI:** 10.1371/journal.pone.0305958

**Published:** 2024-08-22

**Authors:** Furkan Cengiz, Ozkan Isik, Zlatan Bilic, Dario Novak

**Affiliations:** 1 Institute of Health Sciences, Balikesir University, Balikesir, Türkiye; 2 Faculty of Sports Sciences, Balikesir University, Balikesir, Turkiye; 3 Directorate of Sports Sciences Application and Research Center, Balikesir University, Balikesir, Türkiye; 4 Faculty of Kinesiology, University of Zagreb, Zagreb, Croatia; University of Montenegro, MONTENEGRO

## Abstract

It is known that different types of music used during sports performance has different psycho-physiological effects. In this context, this study aimed to reveal the effect of different types of music on ITN test performance in tennis players. A total of 35 recreational tennis players voluntarily participated in the study. In the research, the ITN test performance of tennis players was measured in three different conditions with 48-hour intervals, these being without music, with motivational music, and with sedative music. The Freidman test was used in the analysis of the data that did not show a normal distribution, and the Repeated Measures ANOVA test was used in the analysis of the data that showed a normal distribution. According to the main findings of the study, it was determined that motivational music increased the ITN test performance of tennis players, while sedative music decreased their ITN test performance (p< .05). Additionally, it was determined that motivational music increased the groundstroke depth scores of tennis players, while sedative music decreased groundstroke depth scores (p< .05). In addition to this, it was determined that volley depth, groundstroke accuracy, and serve scores increased through motivational music (p< .05), whereas sedative music had no effect (p> .05). As a result, it was determined that the ITN test performances of tennis players can be increased through the psycho-physiological effect of motivational music. It can therefore be concluded that the use of motivational music during training and matches (between sets and/or time breaks) of tennis players can increase their shooting performance.

## Introduction

A tennis match can be described as an activity characterized by repetitive high-intensity actions by intermittent standardized rest periods. The precision of shots is a crucial skill for achieving success in tennis. Even slight differences in centimeters can determine whether a shot is executed successfully or not [1]. The outcome varies based on whether the ball bounces within the court lines or outside of them. This skill holds the utmost significance for professional players competing at the highest level in tournaments, as the number of errors can greatly impact the final result of a match. Recreational players also find it crucial, as the accurate execution of shots during training or tournaments can greatly enhance their satisfaction and sense of improvement. Performing precise shots at a high level also enhances the range of exercises that can be incorporated into training sessions, thereby providing additional encouragement for recreational players to persist with their training. Tennis encompasses numerous interconnected technical and physical aspects, making it a challenging sport, particularly for beginners due to a lack of coordination, this can also be the case for children who have not developed high-level skills to complete complex actions [1]. To refine tennis shots across players of varying skill levels, it is essential to possess perseverance and engage in a significant number of shot repetitions. Furthermore, it is essential to have a significant amount of time on the tennis court. One of the key things is the motivation that encourages us to train. Other aspects can also motivate us to train, including coaches, practice partners, and individuals in our daily lives such as colleagues or fellow tennis club members. Furthermore, motivational videos, inspiring speeches, or music can have a profound impact on a tennis player’s motivation [2–4]. It can be assumed that recreational players may have lower motivation levels compared to professional players who have devoted their lives to tennis and constantly practice for progress [5]. It is recommended to incorporate multiple factors for recreational players to enhance their motivation for consistent training. One of them is music, which has shown positive effects in previous research on motivation during training [6–11]. These mentioned studies primarily focused on monocyclic activities, where the noise was not a significant disruptive external stimulus. In contrast, tennis demands a considerable amount of focus while playing, and external stimuli like music could significantly affect the performance of tennis players. Listening to music during training can also have various positive effects, as highlighted by the authors in their research, such as improving mood [12, 13], reducing stress [14], and enhancing the rhythm of movement performance [15]. Enhancing the rhythm in movement has the potential to have a positive impact on improving movement and shot positioning. Consequently, this can establish a solid foundation for enhancing overall performance, particularly in tests assessing shot precision. For recreational tennis players who engage in the sport for pleasure a few times a week, and who may not have a high level of physical fitness, listening to music offers a particularly important benefit where they subjectively reduce their perception of effort when compared to training without music [16–19]. Consequently, these players do not experience excessive fatigue or overtraining, but instead maintain a satisfactory level of satisfaction and physical well-being during their tennis training. Considering that tennis is a complex sport and demands a significant level of coordination of the whole body, as well as hand-eye coordination with the game’s dynamic, the impact of music, if beneficial, can enhance the synchronization between movements, consequently improving overall performance. It is worth noting that different tennis players have unique personalities and characters, which become evident during training and especially during competitions. Considering these individual differences, it can be assumed that each player has a preference for a specific genre of music during their leisure time and that this choice influences their everyday activities differently. Furthermore, one could contend that individuals select music based on factors such as the tempo, rhythm, and lyrics that align with their current physical activities. Previous research [20–23] has demonstrated significant correlations between the type of music, particularly its speed and rhythm, and its impact on reducing stress and heart rate. Positive songs with a pleasant rhythm also can have a positive impact on mood and help reduce stress [21, 22]. By increasing positive thinking, it is possible to increase the focus on successful performance, and thus on the results of the performance of shots on the tennis court in various field tests, primarily on the precision of the shot execution. Furthermore, research has indicated that fast music can have positive effects on high-intensity activities like tennis [24, 25]. High-tempo music, defined as music with a tempo of over 120 beats per minute, has been found to stimulate tennis players and enhance their performance [8, 25]. Moreover, prior studies [26] have indicated that during high-intensity activities like HIIT training, incorporating fast-paced music has demonstrated a positive impact on the overall satisfaction levels of tennis players, surpassing the experience of training without any music. Considering the numerous positive effects of listening to music, it is worth exploring its usefulness while playing tennis and how different types of music can impact enhancing performance in tennis. The novelty of this study lies in the analysis of the precision of recreational players’ shots, which is one of the essential skills in tennis. According to the rules of the game, playing music during tennis matches is strictly forbidden. However, tennis academies incorporate music into their practice sessions with professional and recreational players. Listening to music during tennis practice is particularly popular among recreational players during group training sessions for adults and young players. The affect music has on the precision of performance in tennis can be tested by various tests that assess the precision of performance on the tennis court. In this study, the ITN performance test will be used. This study aims to investigate the effects of different music tempos on shot precision among recreational tennis players.

## Materials and methods

### Calculation of the sample size

In order to generalize the research results, a power analysis was performed to determine the sample size. The total number required to find the expectation of a large effect size (f=0.60) to be statistically significant in revealing the effects of different music types on the ITN test performance in tennis players was determined as 30 (α=0.05; 1-β=0.80). Due to the possibility that one or more of the tennis players participating in the research could not complete the research, 35 tennis players were included in this research.

### Research group and ethical approval

Thirty-five licensed adult tennis players ($\bar{X}$ age = 34.29 ± 7.83) who had been playing tennis regularly for at least 2 years voluntarily participated in the research. Their average ITN levels were between ITN7 and ITN9. All participants were medically checked before starting the study. Adult participants were selected based on fitness levels, more specifically those who did not have a condition that had prevented them from exercising in the last six months, did not use any chronic medication, and did not have any skeletal-muscular injuries. It was also determined that the participants had not conformed to a special diet program or any nutritional supplement programs in the last six months. They were also warned against drinking alcohol. Moreover, the tennis players were asked to try and have a good nights sleep before the measurement days. Participants were given detailed information about the purpose and content of the study and signed an informed consent form. Ethical approval for this research was received from Balıkesir University (Decision No: 2021/20) and the research was conducted following the Declaration of Helsinki.

### Experimental desing

Before the ITN tests, all tennis players performed a 10-minute physical and 5-minute technical warm-up, followed by a 30-35-minute ITN test and a 10-minute cool-down exercise. To eliminate the effect of learning, all tennis players practiced the ITN test at the beginning of the study and the obtained scores were not recorded. After familiarization, the ITN test performances of the tennis players were repeated in three different conditions: without music, with motivational music (>120 bpm) and with sedative music (<90 bpm), respectively, with an interval of 72 hours. The same individual fed balls to the tennis players during all tests. Motivational and sedative music selections are left to the individual preferences of the participants. ITN test performance measurements of all the tennis players started on 16/May/2022 and ended on 25/May/2022. All tests were carried out at the same time of day (between 16:00 and 19:00) on each measurement day. All measurements were carried out in an environment suitable for an official tennis match (official court, balls, and rackets) and the air temperature was between 25-27°C. All ITN tests in three different conditions were video recorded.

### ITN test

The ITN test is often used to determine the levels of tennis players at various sports clubs. The ITN On Court Assessment is a tennis skill assessment method developed by the International Tennis Federation. The ITN assessment is made up of the following tasks:

1. *Groundstroke Depth Assessment* - includes a power aspect (10 alternate forehand and backhand ground strokes). The Groundstroke Depth Assessment has been designed to enable players to test their control, depth, and power. Players will receive Double Points if the second bounce is beyond the Bonus Line. Players only receive points for hitting balls into the singles playing area of a tennis court. The player hits 10 balls that are fed to alternate sides, one Forehand, one Backhand, one Forehand, one Backhand, etc. Points are awarded based on where the ball lands on the first and second bounce. When a ball’s first bounce lands anywhere outside the normal singles playing area, it scores zero points.

2. *Groundstroke Accuracy Assessment* - includes a power aspect (6 alternate forehands and backhands down the line and 6 alternative forehands and backhand cross-court). The player should hit each ball down the line. Six balls are fed to alternate sides, (one forehand, one backhand, one forehand, one backhand), etc. The player should hit each ball cross-court. Points are awarded based on where the ball lands on the first and second bounce. When a ball’s first bounce lands anywhere outside the normal singles playing area, it scores zero points.

3. *Volley Depth Assessment* - includes a power aspect (8 alternate forehand and backhand volleys). The player should hit 8 balls that are fed to them to alternate sides, one Forehand, one Backhand, one Forehand, one Backhand, etc. Points are awarded based on where the ball lands on the first and second bounce. When a ball’s first bounce lands anywhere outside the normal singles playing area, it scores zero points.

4. *Serve Assessment* - includes a power aspect (12 serves in total, 3 serves in each target area). The player hits 12 Serves. -Three serves to the wide area of the first service box, three serves to the middle area of the first service box, three serves to the middle area of the second service box and three serves to the wide area of the second service box. Points are awarded based on where the ball lands on the first and second bounce. If the first serve lands anywhere in the correct service box, no second serve is required. If the serve is a let, the serve is replayed. When a ball’s first bounce lands anywhere outside the normal singles playing area, it scores zero points.

5. *Mobility Assessment* - measures the time it takes a player to pick up five tennis balls and return them individually to a specified zone. The score is recorded in seconds. Points are awarded based on the time it takes to complete this task. The faster a player completes the task, the more points awarded [27]. Since the mobility assessment is an assessment that measures the velocity of tennis players, it was not included in the research.

### Reliability of data

The tennis players’ scores during the test were recorded on the ITN test form. To ensure the reliability of the data, three experienced tennis coaches (two 2nd level and one 3rd level) compared the accuracy of the values observed in the video recording with the scores in the test form by examining them one by one, and the coefficient of agreement between the obtained data was tested with Cohen’s Kappa analysis. According to the comparison results, it was seen that the fit coefficient (κ) for variables was found to be 1.0. This result shows that the reliability of the data obtained is in perfect agreement [28].

### Statistical analysis

SPSS was used to analyze the data obtained. Mean, standard deviation, and median were used as descriptive statistics of the data. The normality of the data was tested with the Shapiro-Wilk Test and the Freidman test was used for variables with a p< 0.05 value. Dunn’s post-hoc test was used to determine the differences between groups in data that did not show a parametric distribution, and the Repeated Measures ANOVA test was used for variables with a p> 0.05 value. LSD post-hoc test was used to determine the differences between groups in data showing parametric distribution. Significance was set at p< .05.

## Results

According to the Shapiro-Wilk test results of the normality test of the data obtained, non-parametric tests (χ2 value) were used for variables with p< .05 level and parametric tests (F value) were used for variables with p> .05 level (Table 1).

**Table 1:** Normality test results for ITN test variables.

ITN Test Variables	Conditions	Statistics	p
**Groundstroke Depth**	Without Music	.963	.287
	Motivational Music	.966	.339
	Sedative Music	.975	.584
**Volley Depth**	Without Music	.883	.001*
	Motivational Music	.982	.825
	Sedative Music	.925	.020*
**Groundstroke Accuracy**	Without Music	.918	.013*
	Motivational Music	.980	.770
	Sedative Music	.909	.007*
**Serve**	Without Music	.923	.018*
	Motivational Music	.948	.098
	Sedative Music	.852	.001*
**ITN Score**	Without Music	.942	.065
	Motivational Music	.948	.097
	Sedative Music	.963	.285

*p< .05

When the groundstroke depth scores of tennis players in three different conditions were compared, it was observed that the average scores obtained from each condition were different from each other (p< .05). Accordingly, the highest groundstroke depth score was achieved in the condition with motivational music (Table 2).

**Table 2:** The comparison of groundstroke depth scores performed in three different conditions.

Groundstroke Depth Scores	$\bar{X}$±S.D.	F	p
Without Music	34.11±9.48^b^	121.619	.001*
Motivational Music	52.89±8.79^a^		
Sedative Music	31.77±7.13^c^		

*p< .05; abc: Differences between groups are represented by different letters.

N: 35

When tennis players’ volley depth scores were compared in three different conditions, it was determined that there was a statistically significant difference between the volley depth scores in the condition with motivational music and the volley depth scores in the condition without music and with sedative music (p< .05). On the other hand, it was determined that there was no statistically significant difference between the volley depth scores in conditions with sedative music and without music (p> .05; Table 3).

**Table 3:** The comparison of volley depth scores performed in three different conditions.

Volley Depth Scores	$\bar{X}$±S.D.	(Q1-Q3)	Q2 (Medyan)	χ2	p
Without Music	22.94±9.68	(16.00-27.00)	20.00^b^	44.143	.001*
Motivational Music	38.46±8.85	(30.00-45.00)	39.00^a^		
Sedative Music	21.94±7.75	(16.00-26.00)	22.00^b^		

*p< .05; ab: Differences between groups are represented by different letters.

N:35

When tennis players’ groundstroke accuracy scores were compared in three different conditions, it was determined that there was a statistically significant difference between the groundstroke accuracy scores in the condition with motivational music and the groundstroke accuracy scores in the condition without music and with sedative music (p< .05). On the other hand, it was determined that there was no statistically significant difference between the groundstroke accuracy scores in conditions with sedative music and without music (p> .05; Table 4).

**Table 4:** The comparison of groundstroke accuracy scores performed in three different conditions.

Groundstroke Accuracy	$\bar{X}$±S.D.	(Q1-Q3)	Q2 (Medyan)	χ2	p
Without Music	22.66±8.26	(16.00-27.00)	21.00^b^	54.136	.001*
Motivational Music	46.91±8.36	(43.00-52.00)	48.00^a^		
Sedative Music	22.77±6.36	(18.00-26.00)	23.00^b^		

*p< .05; ab: Differences between groups are represented by different letters.

N:35

When tennis players’ serve scores were compared in three different conditions, it was determined that there was a statistically significant difference between the serve scores in the condition with motivational music and the serve scores in the condition without music and with sedative music (p< .05). On the other hand, it was determined that there was no statistically significant difference between the serve scores in conditions with sedative music and without music (p> .05; Table 5).

**Table 5:** The comparison of serve scores performed in three different conditions.

Serve Scores	$\bar{X}$±S.D.	(Q1-Q3)	Q2 (Medyan)	χ2	p
Without Music	13.49±5.97	(8.00-16.00)	12.00^b^	40.187	.001*
Motivational Music	23.77±5.48	(20.00-28.00)	26.00^a^		
Sedative Music	13.03±5.10	(10.00-14.00)	12.00^b^		

*p< .05; ab: Differences between groups are represented by different letters.

N:35

When the ITN scores of tennis players in three different conditions were compared, it was observed that the average scores obtained from each condition were different from each other (p< .05). Accordingly, the highest ITN score was achieved in the condition with motivational music (Table 6).

**Table 6:** The comparison of ITN scores performed in three different conditions.

ITN Scores	$\bar{X}$±S.D.	F	p
Without Music	93.20±21.46^b^	352.823	.001*
Motivational Music	162.03±21.80^a^		
Sedative Music	89.51±16.22^c^		

*p< .05; abc: Differences between groups are represented by different letters.

N:35

## Discussion

The objective of this research was to examine how different music tempos affect the precision of shots in recreational tennis players. The findings revealed that playing motivational music with a tempo of 120 beats per minute while taking the ITN test resulted in a positive influence on the precision performance of recreational players. The study’s key discovery is that playing motivating music during specific tennis tasks in the ITN test leads to a significant improvement in performance compared to listening to sedative music or no music at all. This finding highlights the importance of music in enhancing performance in sports. A prior study has corroborated these findings, indicating that when athletes perform high-intensity activities such as tennis, motivating music is the optimal selection for enhancing outcomes [29]. However, contrasting findings in similar studies regarding the impact of motivational music can be attributed to variations in participants’ levels of motivation, as well as disparities in the lyrics, volume, and melody of the music used [8, 30]. Previous research has also indicated [31] that engaging in resistance exercise while listening to motivating music with a tempo of 120 beats per minute can lower lactate levels and modify cortisol levels in athletes, which can ultimately contribute to improving their overall performance. These alterations in biochemical markers can potentially delay or alleviate fatigue. Considering that the tests conducted in this study may induce fatigue, it can be inferred that motivating music could postpone the onset or perception of fatigue. Furthermore, the outcomes can be elucidated by the discoveries from previous studies [32] in which inspirational music exhibited a favorable impact on visual cognition, enhanced focus, and motor coordination during the execution of diverse motor assessments. In the realm of tennis, maintaining a high level of attentiveness towards the approaching ball is of utmost significance as it determines the opportune moment to hit the ball with utmost precision [33]. The findings from additional studies [12, 34, 35] demonstrate the positive impact motivational music has on enhancing starting speed and reaction speed, of which can provide valuable insights into the outcomes of this research. Undoubtedly, improved starting speed and reaction speed will greatly enhance the ability to perform tasks in the ITN test, thereby creating a stronger foundation for achieving higher levels of shot precision from a technical standpoint. The optimal outcomes across all measured factors are achieved through the use of motivational music. These outcomes hold considerable practical significance, considering the additional advantages associated with the music. Enhanced accuracy in fundamental shots will undoubtedly heighten the enjoyment of recreational players during their training sessions. Building upon the positive outcomes in precision parameters, it becomes feasible to design and implement training programs that offer greater variety and complexity, thereby fostering even more motivation among recreational players. Playing motivating music during the ITN test can greatly enhance the accuracy and quality of shots, including the forehand, backhand, volley, and serve. Even if players who undergo the test opt to compete in tennis tournaments that prohibit music during the game, they can still attain positive outcomes by training with motivational music. The analysis of all variables in conditions without music and with sedative music reveals that the outcomes are comparable, indicating no significant disparity. This is particularly evident in groundstroke depth scores (34.11/31.77), volley depth (22.94/21.94), groundstroke accuracy (22.66/22.77), and serve (13.49/13.03). Additionally, the ITN scores variable also demonstrates statistically insignificant results, although it suggests that players perform better without music as opposed to sedative music. It can be presumed that sedative music has excessively calming and demotivating impacts on tennis players during their performance, thereby failing to stimulate them to achieve their utmost potential.

Although a silent environment is created during an official competition for athletes can focus maximum, it is a known fact that they prefer to use motivational music and stay motivated throughout training. Considering that the examined recreational players, are not involved in professional competition, and the majority of them do not participate in tournaments at all but solely focus on training sessions, the findings of this study hold significant practical relevance in this particular scenario. Coaches can incorporate motivational music throughout all phases of training. It is expected that this will enhance the level of success across all training segments, not just limited to ITN tests. This includes the warm-up phase at the beginning of the training, the core part of the training, and the final phase. Previous research [36] has indicated that motivational music has a beneficial effect on the execution of a golf swing, which is classified as a closed-chain motor skill. This finding can be linked with the performance of the service in the ITN test within the same study, which is also categorized as a closed-chain motor skill shot. The outcomes demonstrate that listening to motivational music leads to significantly improved results compared to conditions involving sedative music or no music at all.

This study has several limitations and directions for further research. It would be intriguing to explore this topic among professional tennis players, who typically train more regularly without music than with music due to the restrictions during official matches. Future studies could investigate the impact of motivational music on their ability to execute basic shots during training, as well as whether this benefit can translate to actual game conditions without the presence of music. The subjective assessment of the load and satisfaction of performing tasks was not measured in this study. Additionally, there was no variation in the results based on the gender of the participants, and no discernible impact on the competitive performance of recreational players was observed. The study did not control the volume of music, which could potentially impact performance due to the influence of song lyrics and melodies.

## Conclusion

To summarize, the outcomes of this study suggest that incorporating motivational music can be a valuable strategy for enhancing the performance of recreational tennis players during specific tasks. Allowing players to listen to their preferred music at a tempo of 120 beats per minute during training sessions may lead to improved performance in various tests. Despite the limitations of this study, the results indicate that motivational music interventions have the potential to enhance the performance level of play for recreational tennis players. Additionally, coaches should consider integrating these findings into their training sessions to optimize both training and performance outcomes. This study serves as a solid foundation for further discussion and future research, which could ultimately contribute to these findings. However, it is important to note that additional research is required to validate these results.

## Supporting information

S1 Dataset (SAV)
